# Financial difficulty in the medical profession

**DOI:** 10.1177/01410768231172151

**Published:** 2023-05-22

**Authors:** Asta Medisauskaite, Rowena Viney, Antonia Rich, Kirsty Alexander, Milou Silkens, Laura Knight, David Harrison, Paul Crampton, Ann Griffin

**Affiliations:** 1 UCL Medical School, 4919 University College London, Research Department of Medical Education, London, WC1E 6BT, UK; 2 Hull York Medical School, Health Professions Education Unit, York, YO10 5DD, UK; *Joint first authors.

Medicine is a relatively well-paid profession, with base salaries substantially over the minimum wage. However, students leave medical school with considerable debt and a growing proportion of medical students and doctors struggle with financial worries and/or difficulties. This commentary sheds light on the types and causes of financial difficulties that medical students and doctors face, highlighting that it can happen for a range of reasons, all of which should be considered when developing professional financial support systems. Understanding the context of financial difficulties within the profession is important for a variety of reasons, including maximising individual practitioner wellbeing and workforce planning. For example, concerns continue to be raised about the underfunding of medical students leading them to ‘*interrupt their studies, fail exams, or drop out of medical school entirely due to financial neglect by this government*’.^1^ Furthermore, a junior doctor’s income is 26% lower in real terms now than in 2008^2,3^ and may well be contributing to the ongoing retention crisis within the profession. Indeed, in the current context of a cost-of-living crisis in the UK, and with 98% of junior doctors voting for strike action in 2023 over pay issues and working conditions (with a ballot turnout of 77%),^4^ it is all the more important to understand and highlight the types and causes of difficulty, particularly if important efforts to widen access to medicine are to continue.

Our observations are based on a literature review and interviews that were part of a larger study funded by the Royal Medical Benevolent Fund (approved by UCL Research Ethics Committee, ref. 13311/003). We conducted a narrative review of academic and grey literature published between 2011 and 2021 that concerned the financial need of medical students and doctors in the UK education and healthcare context. Searches in four academic databases (PsychInfo, SCOPUS, Web of Science, ProQuest), websites of 39 relevant medical and educational organisations (including the Royal Colleges, British Medical Association, General Medical Council and student loan organisations) and citation searching led to 47 results being included in the final synthesis from 1491 initial hits (see Table 1 for reference list). Semi-structured interviews were conducted with 25 experts – people who have experience of working with doctors and/or medical students who are in financial need (e.g. student support, occupational health, charities that offer support). Data about groups of people who the experts perceived to be at risk of financial need were extracted from the interviews.

**Table 1.** Papers identified through the literature review.

British Medical Association. *Disability in the medical profession: Survey findings 2020*. Report, 2020. BMA website.
The RTK. *Identifying unmet needs from the General Medical Council Gateways to the Professions guidance.* London: General Medical Council. Report, 2018.
Knight C. Fund in memory of medic will help those at risk of suicide. *Evening Chronicle*, 20 March 2018; 4.
Patel RS, Tarrant C, Bonas S, et al. Medical students’ personal experience of high-stakes failure: Case studies using interpretative phenomenological analysis. *BMC Medical Education* 2015; 15: 1–9.
O’Hara V. Belfast doctor back from brink after battle with alcoholism hails medics ‘support group’. *Belfast Telegraph*, 23 March 2016; 18.
British Medical Association. Briefing on COVID-19 and childcare. Briefing, 2020. BMA website.
British Medical Association. Childcare support for doctors must improve. News item, 20 May 2020. BMA website.
Cleland JA, Nicholson S, Kelly N, et al. Taking context seriously: Explaining widening access policy enactments in UK medical schools. *Medical Education* 2015; 49: 25-35.
Cleland JA, Dowell J, McLachlan J, et al. *Identifying best practice in the selection of medical students: literature review and interview survey.* London: General Medical Council. Report, 2012.
Krstić C, Krstić L, Tulloch A, et al. The experience of widening participation students in undergraduate medical education in the UK: A qualitative systematic review. *Medical Teacher* 2021; 43: 1044-1053.
Vaughan S. *Medical students’ experience and achievement: the effect of ethnicity and social networks.* Manchester: University of Manchester. Thesis: 2013.
Anane M, Curtis S. An exploration of the implications of employment for medical students. A comparison of widening participation students to traditional entry students. In: *Abstracts Book ASM 2019*. Edinburgh: Association for the Study of Medical Education. Abstract, 2019.
Raven PW. If doctors can train part time, why not medical students? *BMJ* 2014; 349: g4897.
Cohen D, Winstanley S, Palmer P, et al. *Factors that impact on medical student wellbeing – perspectives of risks*. London: General Medical Council. Report, 2013.
Bassett AM, Brosnan C, Southgate E, et al. The experiences of medical students from First-in-Family (FiF) university backgrounds: a Bourdieusian perspective from one English medical school*. Research in Post-Compulsory Education* 2019; 24: 331–355.
Claridge H, Ussher M. Does financial support for medical students from low income families make a difference? A qualitative evaluation. *BMC Medical Education* 2019; 19: 1–8.
British Medical Association. BMA NI medical students committee appeals to Health Minister over covid payment. Press release, 5 February 2021. BMA website.
Woolf K, Rich A, Viney R, et al. *Fair Training Pathways for All: Understanding Experiences of Progression*. London: General Medical Council. Report, 2016.
Coyle D. Bank won’ t fund my medical course in England. *Irish Times* 17 July 2012; 6.
British Medical Association. Clinical excellence reworked - the tax bill that put a consultant’s future at risk. News item, 25 October 2019. BMA website.
British Medical Association. On the ground: pay protection. News item, 11 January 2021. BMA website.
British Medical Association. On the ground: paid leave in question. News item, 16 May 2021. BMA website.
Royal College of Anaesthetists. Almost three quarters of anaesthetists in training subjected to late or inaccurate salary payments by NHS hospitals. News item, 16 June 2018. Royal College of Anaesthetists website.
Royal College of General Practitioners. RCGP dismayed and deeply concerned after hundreds of trainee GPs are left waiting for pay. Press release, 3 November 2017. Royal College of General Practitioners website.
Power B. NHS talks to new surgery operator. *Cornish Guardian* 20 August 2014; 19.
Power B. Doctors quit NHS. *Cornish Guardian* 13 August 2014; 1.
Riley R, Spiers J, Buszewicz M, et al. What are the sources of stress and distress for general practitioners working in England? A qualitative study. *BMJ Open* 2018; 8: e017361.
Community Research. *The effects of having restrictions on practice or warnings*. London: General Medical Council. Report, 2015.
British Medical Association. Compensation claim for doctors on paused rotas. News item, 30 July 2020. BMA website.
British Medical Association. BMA calls on Chancellor to provide enhanced death in service cover to all frontline NHS staff. Press release, 1 April 2020. BMA website.
Zhou AY, Panagioti M, Esmail A, et al. Factors Associated with Burnout and Stress in Trainee Physicians: A Systematic Review and Meta-analysis. *JAMA Network Open* 2020; 3: 1-16.
Khadjooi K, Scott P, Jones L. What is the impact of pregnancy and parenthood on studying medicine? Exploring attitudes and experiences of medical students. *Journal of the Royal College of Physicians of Edinburgh* 2012; 42: 106-110.
Curtis S, Smith D. A comparison of undergraduate outcomes for students from gateway courses and standard entry medicine courses. *BMC Medical Education* 2020; 20: 1-14.
Vogan CL, McKimm J, da Silva AL, et al. Twelve tips for providing effective student support in undergraduate medical education. *Medical Teacher* 2014; 36: 480-485.
Nunez-Mulder L. Medical students consider abandoning degree because of financial pressures, survey finds. *BMJ* 2018; 363: k4990.
Horsfall, S. *Doctors who Commit Suicide while under GMC Fitness to Practise Investigation.* London: General Medical Council. Report, 2014.

Our findings showed that all medical students and doctors are at risk of experiencing financial difficulties in their medical career. Some groups, however, are particularly vulnerable to financial difficulty:

- *Medical students*, due to study expenses, high living costs and limited possibilities for additional paid work due to the high workload on their courses. This was particularly relevant for students from widening participation backgrounds and overseas students;
- *Medical trainees*, due to student debt, vulnerability to financial mismanagement and training costs;
- *Doctors from overseas* (international medical graduates and refugee doctors/asylum seekers), due to having high expenses setting-up in the UK and being unfamiliar with UK systems;
- *Doctors out of work* (e.g. under GMC investigation) *or with non-substantive employment* (e.g. locum), due to their jobs being less secure;
- *Medical students and doctors affected by the pandemic*, due to being unable to work because of shielding, developing long-COVID or students not being able to find work outside medicine due to restrictions in sectors such as retail and hospitality.

The challenges that can lead to financial hardship for medical students and doctors are summarised in five key pathways (Figure 1). Financial difficulties can arise from an individual pathway or from a combination of multiple pathways.

**Figure 1.**
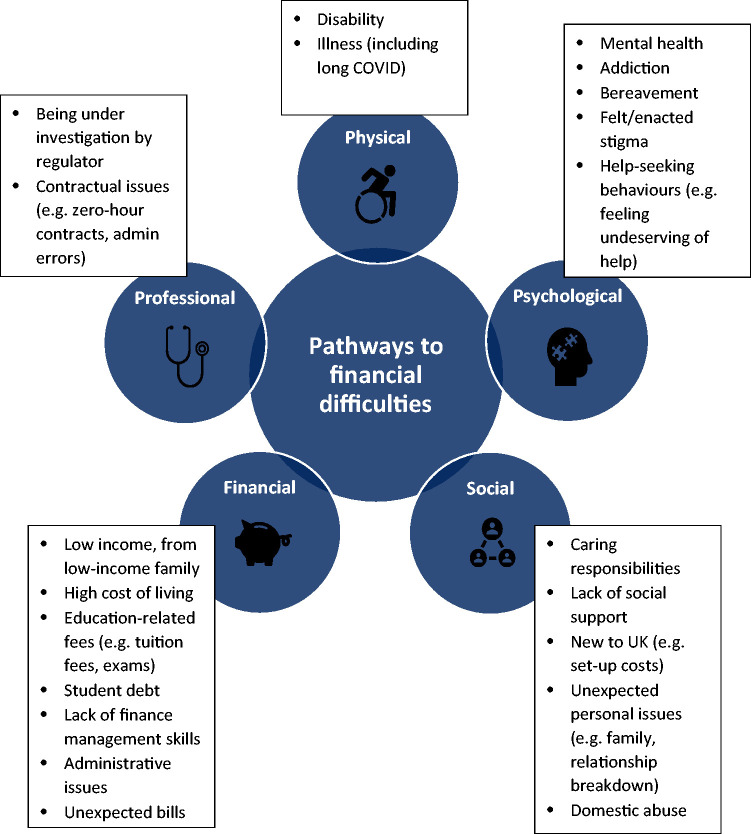
Pathways to financial difficulties.

## The physical pathway

Illness or disability can put a financial strain on medical students and doctors due to having to study or work less-than-full-time,^5^ having difficulty accessing financial support^6^ or lack of awareness of available support (interviews). It can be particularly challenging if illness is unexpected.^7,8^ Those who are very ill might find seeking support too challenging and for that reason avoid it (interviews).

## The psychological pathway

Mental health issues contribute to financial difficulty due to time away from practice (interviews and O’Hara^9^). Those with addiction issues can fall into spiralling behaviours, experience breakdown of relationships and loss of work (interviews). Additionally, negative attitudes to seeking help are a powerful barrier compounding financial challenges; acknowledging needing help can feel like admitting to failure, and medical students and doctors fear the impact of this on their career and can also feel undeserving of help (interviews). Feelings of stigma and shame around experiencing financial difficulties can compound the situation, by leading to or exacerbating mental health issues, and being a further barrier to seeking help before reaching crisis point (interviews).

## The social pathway

Numerous social factors can contribute to financial difficulties. Caring responsibilities can lead to financial need. Doctors with dependent children bear the extra cost of childcare,^10,11^ and carers can experience financial difficulties if they are unable to work fulltime (interviews).

Social systems are important for financial stability. Having a lack of social or family support is a risk factor for students,^12–16^ particularly highlighted in the context of widening participation students whose families are less likely to be able to provide financial support (interviews). Those in abusive relationships (i.e. domestic abuse victims) are particularly financially vulnerable (interviews).

Students are especially vulnerable to circumstantial changes impacting their financial stability (e.g. relationship breakdowns leading to extra expenses)^7,8^ as there is a lack of available grants offering financial support to them in this context (interviews).

## The financial pathway

Students might need paid work to meet their living costs.^16^ However, there is little time for paid work during term time due to the courses’ high workload. This, together with high course fees, can cause medical students to get into financial difficulty.^17^ Zero-hour contracts could provide more flexibility; i.e. 38.7% of those on zero-hour contracts in the UK in 2023 were aged 16–24 years.^18^ However, the lack of security which comes with zero-hour contracts can make it challenging to plan finances.

Student loans available for medical students are the same as those offered to students on other courses^19^; this does not take into account that the course and the terms are longer, meaning that medical students need money to pay for their studies and expenses for longer than other students.^20,21^

Foundation year doctors can have student debt as large as £100,000, which can take decades to repay with interest on student loans currently up to 6.9% or the Retail Price Index,^22^ and they may lack the skills needed to handle their financial situation (interviews). Students from low-income backgrounds and/or not in receipt of familial financial support may be especially vulnerable during their transition to the foundation years, as they could encounter expenses due to relocating and purchasing appropriate clothing before being paid (interviews).

Medical training comes with considerable work-related expenses. Similar to some other professional training, medical students have to pay for professional clothing and commuting to placements, and for extra-curricular activities that they are expected to do to improve their curriculum vitae, e.g. conferences.^19–21,23^ Trainees need to pay for courses (interviews) and exam fees, and may have to pay to sit exams multiple times.^24^ As an example, surgical trainees spent between £20,000 and £71,431 depending on their sub-specialty to achieve the mandatory requirements for completion of training in 2017.^25^

Overseas students have to pay higher fees but have fewer opportunities for financial support in the UK^10^ or from their home countries.^26^ For overseas doctors (international medical graduates, refugees and asylum seekers), high expenses setting up, visa costs and limited available resources might contribute to financial challenges (interviews).

Administrative issues can lead to financial difficulties, including changes in tax rules leading to lower income^27^ and administrative errors leading to delay or loss of pay.^28–31^

## The professional pathway

Financial responsibilities related to the workplace might lead to financial difficulties even for more established practitioners, for example general practice partners may have to take on financial responsibility for their practices.^32–34^ Doctors who work in private care can also experience financial difficulties if they have pay-as-you-go contracts (interviews): if the work dries up, so does the income. Locums might also experience difficulties as locum work is less secure (interviews). Finally, doctors who have fitness to practise warnings, undertakings or conditions may experience financial difficulties if they are limited to working fewer hours or in different roles or being out of work altogether (interviews and General Medical Council^35^).

It is also important to add that disruption caused by the pandemic created additional financial challenges. For example, better-paid rotations being cancelled meant that some trainees’ income was less than expected (higher pay through out-of-hours commitments, intensity payments and other enhancements)^36^ and government restrictions on hospitality and retail sectors meant less availability of part-time work for medical students.^37^ The long-term impact of the pandemic should also be acknowledged: for example, doctors suffering from long-COVID are likely to be out of work for longer than 12 months which can jeopardise their contracts (interviews).

Financial challenges can have a significant negative impact on medical students and doctors, specifically their mental health and wellbeing,^16,38^ quality of life^8,15,39^ and progression through education,^40–42^ and can even impact the medical workforce.^11,27,43^

## Conclusion

The potential to experience financial difficulty is widespread throughout a person’s medical career, from medical school through to full qualification. Our overwhelming impression, given the strength of the evidence in the literature review and the varied stories from our interview participants, is that this can happen to anyone and can arise from a range of sources: physical, psychological, social, financial and professional. There is a need for wide-ranging financial systems to support the profession. There is a lot of shame and stigma still associated with financial need, which can affect people’s willingness to seek support before reaching crisis-point. More research on the levels of financial need within the medical community will allow us to see just how much need there is, helping to plan and target the support that is available from organisations. More open discussion about financial need will also potentially reduce the stigma around this topic and inform people about managing their finances.

## Declarations

### Guarantor

All authors.

### Provenance

Not commissioned; editorial review.
